# Leveraging Immersive Virtual Reality to Prevent Child Injuries in the Home: Proof-of-Concept Study

**DOI:** 10.2196/90006

**Published:** 2026-09-30

**Authors:** Melissa C Osborne, Lei Zhang, Allison Garefino, Reece Freeman

**Affiliations:** 1 Wellstar School of Nursing, Wellstar College of Health and Human Services, Kennesaw State University, 520 Parliament Garden Way NW, MD 4102, Kennesaw, GA, 30144, United States, 1 4705786061; 2 Department of Software Engineering and Game Development, College of Computing and Software Engineering, Kennesaw State University, Kennesaw, GA, United States; 3 Wellstar College of Health and Human Services, Kennesaw State University, Kennesaw, GA, United States

**Keywords:** injury prevention, child health, immersive virtual reality, parent behavior, parenting programs, unintentional injury, child safety

## Abstract

**Background:**

Injury is the leading cause of death among US children, with over 2700 deaths due to unintentional injuries among children aged <6 years in 2023. Many of these deaths occur in the home and involve addressable safety hazards. Evidence-based parenting programs (EBPPs), in which parents work with a provider to learn parenting skills, offer an infrastructure for disseminating home safety content. However, few programs have structured modules on home safety, with most home safety education coming from printed materials or websites. Immersive virtual reality (IVR) allows participants to engage in activities in a simulated, risk-free environment. IVR’s technological affordances create a training environment that supports experiential learning and helps learners internalize and transfer knowledge more effectively to real-world situations.

**Objective:**

This study aimed to (1) design an IVR home safety program for parents of children aged <6 years in line with our conceptual model, (2) obtain feedback from parents and EBPP providers on the initial design, and (3) test an initial prototype of the program with parents.

**Methods:**

The prototype was developed using human-centered design principles. The team first identified key components for program content using existing research and theory. Qualitative data were then collected from EBPP provider interviews (n=5) and focus groups with parents (n=7) regarding implementation constraints, program content, barriers/facilitators for engagement, and feedback on the use of IVR. Thematic analysis was used to identify themes from the qualitative data. The initial prototype was then pilot tested with parents of children aged <6 years (n=5).

**Results:**

The IVR program addresses knowledge and skill enhancement in three key areas: (1) home safety hazard identification, (2) hazard removal/mitigation, and (3) parental supervision. Using surveillance data, we identified leading causes of unintentional injuries in the home environment among US children aged <6 years, including sleep-related deaths, firearm injuries, and drowning. Hazards and corresponding tasks in the IVR program were developed for these leading causes. Findings from interviews with providers indicate that they are open to the use of IVR in EBPPs and envision uses for both group and individual settings. They expressed concerns about the cost of incorporating IVR into their usual programs. Parents were interested in IVR, especially if presented in brief sessions. They also noted a desire for the virtual environment to look realistic and “lived in.”

**Conclusions:**

Currently, home safety information is delivered in a didactic or pamphlet-driven format in most EBPPs, which is not ideal for behavior change. This IVR home safety program will provide unique benefits in training, including (1) real-world simulations, (2) hands-on learning, (3) a risk-free environment, and (4) IVR scenario-based assessment. This study provides support for the use of IVR in engaging parents in home safety education and presents considerations for future research.

## Introduction

### Background on Child Injury

Injury is a major public health issue faced by children in the United States. In 2023, over 3500 children aged 0-5 years died due to injury, amounting to 226,385 years of potential life lost. In addition to fatal injuries in this age group, there were almost 2 million nonfatal injuries that required an emergency department visit [1]. Over 3 quarters of fatalities (77.3%) among children aged 0‐5 years in 2023 were due to unintentional injuries, and these injuries predominantly occur in the home environment [2-4]. Common types of unintentional home injuries associated with the leading causes of death among young children include drowning, suffocation, poisoning, burns and scalds, falls and tip-over incidents, and firearm injuries [1].

### Child Home Injury Prevention

Parents and other primary caregivers (referred to in this paper simply as “parents”) are key to the prevention of home injuries among young children. Fundamental elements of injury prevention include parents’ ability to identify safety hazards, adequately address hazards, and provide proper supervision. Parents’ knowledge of home safety is associated with reductions in observed home safety hazards [5]. Additionally, children whose parents adopt unsafe behaviors in terms of failing to address hazards and provide adequate supervision are more likely to sustain injuries [6]. Supervision is an especially important element of child injury prevention. Research has found this to be a stronger contributor to child injury prevention than simply identifying and addressing hazards [7,8]. One study noted that parents’ proximity “beyond reach” was associated with serious injuries in young children [8].

Efforts exist to educate parents on home safety in order to prevent child injury. For instance, parents may receive written and/or verbal patient education from their pediatrician at child well visits [9]. There are also organizations such as Safe Kids Worldwide [10] that connect parents with safety devices through their local health department and provide home safety and child injury prevention information online and on social media. Additionally, each year over 280,000 families participate in evidence-based parenting programs (EBPPs), which involve parents meeting with a trained program provider in an individual or group setting to learn parenting skills [11]. These programs typically focus on parent-child interaction skills for parents of young children, and many incorporate home safety in some way. However, home safety information delivered through EBPPs is primarily didactic, involving written materials and/or videos with home safety and child injury prevention information, with home safety devices provided in some instances [12]. A notable exception is SafeCare, which includes a dedicated home safety module with opportunities for home assessment by the provider, parent practice, and feedback [13].

### Digital Health and Child Injury Prevention

Digital health tools have been used as a way to engage parents and enhance learning on home injury prevention. To date, this has primarily involved screen-based applications or games. A serious game developed to teach home safety skills was found to be feasible and acceptable among teenage mothers, and their reaction time was faster when they had the opportunity to play the game multiple times [14,15]. This game involved hazard identification in a screen-based virtual home environment and required mothers to maintain focus as distractions such as alarms were deployed periodically in the game [14]. Other serious games and screen-based programs have been deemed feasible and acceptable by parents learning home safety skills [16-18].

Immersive virtual reality (IVR) engages parents and enhances learning beyond what is possible through screen-based interactions. IVR incorporates immersion, embodiment, sense of presence, and interactivity, which have been positively associated with user engagement and learning outcomes [19,20]. Furthermore, IVR has been found to be effective in increasing procedural knowledge [21]. This is especially applicable to home safety education for parents, as much of the knowledge involves understanding the skills necessary and the steps required to create a safer environment. Using IVR, parents can practice skills in a virtual home environment, receiving feedback in real time. Rehearsing skills in a safe, controlled environment allows parents to refine their responses to improve the likelihood of success in the physical world during real-life scenarios.

### Study Aims

The goals of this study included (1) designing an IVR home safety program for parents of children aged 0‐5 years intended for dissemination through EBPPs, (2) developing a prototype of the program, and (3) testing the feasibility and usability of the program with parents.

## Methods

### Overview

The IVR program was developed using principles of human-centered design in a discover, design/build, and test framework [22]. In Phase 1, we conducted preliminary work to identify key content areas and obtained feedback from parents on the initial design. We then developed the prototype and tested it in Phase 2. Each phase is described in more detail below. All data collection from study participants took place between November 2024 and May 2025.

### Ethical Considerations

This study was approved by the Kennesaw State University Institutional Review Board (IRB-FY24-579).

### Discover and Design: Phase 1

#### Overview

We first developed a conceptual model for the IVR home safety program (see Figure 1). It is important to note that the goal in this study was not to test this model but to provide a theoretical foundation for the program. The model integrates components of the behavior change wheel [23] and the cognitive affective model of immersive learning (CAMIL) [24]. The behavior change wheel is centered around a framework that states that capability, opportunity, and motivation lead to behavior (the COM-B system) [23]. Our model incorporates elements of capability and motivation, as this best reflects our individual-level intervention. CAMIL is a theoretical model for understanding the mechanisms by which education and training through IVR are related to learning outcomes [24].

**Figure 1.**
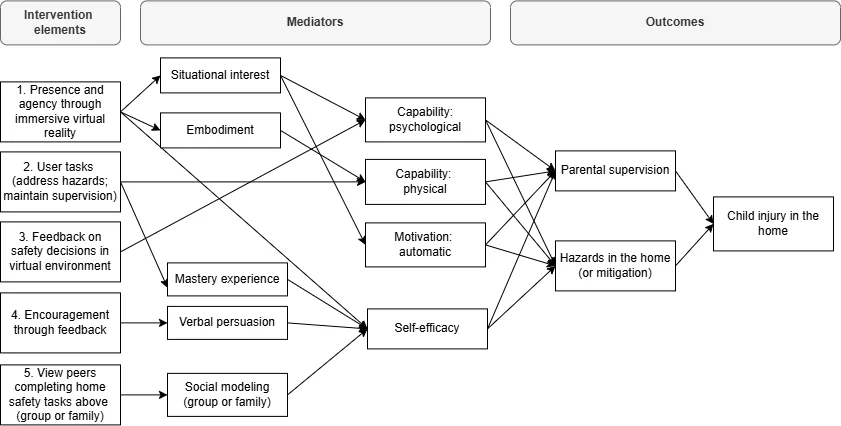
Conceptual model of ParentSHIELD-VR. Mediators are constructs from the behavior change wheel and the cognitive affective model of immersive learning.

The intervention elements are proposed to ultimately lead to the outcomes of interest (ie, parental supervision and home safety hazards and child injury) through several mediators. Figure 1 depicts these relationships. We will now describe how we propose that each intervention element is related to proximal and distal mediators and, ultimately, to the outcomes of interest. We will describe the proposed paths for each intervention element (numbered in Figure 1). First, the model posits that the sense of presence and agency afforded through IVR (Element 1) leads to increased situational interest, embodiment, and self-efficacy. These proximal mediators lead to effects on distal mediators. Specifically, increased situational interest subsequently leads to increased psychological capability (ie, knowledge) [25] and motivation [26-28]. Embodiment leads to increases in physical capability (ie, skills) [21], which has favorable impacts on the outcomes of interest. Next, the model posits that the user tasks in the program (Element 2, ie, identifying and addressing hazards; maintaining proper supervision in the midst of distractions) favorably impact outcomes through increased physical capability (ie, skills) [23]. Feedback provided to users after they address safety hazards in the virtual environment includes facts or additional context around each hazard (Element 3), and this impacts outcomes through increased psychological capability. Similarly, encouraging statements within the feedback (Element 4) affect home safety outcomes through increased self-efficacy [29]. The program is intended for use both in EBPPs that meet with families individually and those that meet in a group setting. Parents meeting in a group setting could watch other parents engaging with the program through the “cast to screen” feature (Element 5). This feature could also be used for EBPP participants who meet as a family with their provider (eg, one parent watching the other parent or caregiver engaging with the program). These situations would offer an opportunity for social modeling, which would impact self-efficacy [29]. Further details on the conceptual model for the program can be found in Figure 1.

As part of Phase 1, we also identified tasks for inclusion in the program. Our team reviewed the literature and publicly available data sources to identify key content areas. We decided to focus the program’s content areas on leading causes of death related to home injury among children aged 0‐5 years. As such, we used data from the Centers for Disease Control and Prevention to identify the leading causes of injury in this age group [1] and considered those that occur in a home environment. The initial set of user tasks designed by the research team related to these leading causes as well as hazards of increasing concern (eg, vaping devices), as identified in recent peer-reviewed literature [30]. We included tasks related to the following hazard categories: poisons, burn/scalds, sharp objects, tip-over/crush, falls, firearms, strangulation, suffocation, and drowning.

#### Provider Interviews

After creating the user tasks and the initial program design in a rough prototype, we conducted qualitative interviews with EBPP providers and focus groups with parents. Specifically, we sought to recruit 5 EBPP providers based on available seed funding resources (ie, funding and timeline). While we would have preferred a sample size of 6‐12, based on recommendations for qualitative interviews [31], we decided to maximize the number of parent voices included in the project while obtaining as much feedback as possible from providers. Inclusion criteria were providers who were currently delivering an EBPP, had been doing so for at least 1 year, and had the technology necessary to participate in a virtual interview through video conferencing software. Administrators from local social service agencies that deliver EBPPs were contacted via email with a description of the study opportunity and a recruitment flyer. They were asked to distribute the opportunity to their providers. Interested providers were instructed to contact the principal investigator (PI; MCO) via email to confirm eligibility. Once eligibility was confirmed, the provider scheduled a virtual qualitative interview with the PI. Each interview took approximately 60 minutes, and providers received a US $35 gift card as compensation.

Qualitative interviews were conducted using an interview protocol developed by the research team. The interview began with the consent process, followed by questions about the provider’s current experiences delivering home safety information to parents (eg, current content provided, areas of interest to families, parents’ receptivity to safety education) and their current use of technology in program delivery. We then presented the provider with a description of the overall program concept, user tasks, and screenshots of the rough prototype. We also shared the proposed name of the program: parent safe home immersive education and learning development–VR (ParentSHIELD-VR). Following this presentation, the provider was given an opportunity to share feedback verbally. We then conducted a Roses, Thorns, and Buds activity [32] in which providers were given a link to a shared Google Sheet where they listed aspects of the program that they liked, things they did not like, and things they would change. Providers were given 5‐10 minutes to provide feedback in this way and then were given the opportunity to expand on any written feedback. Finally, providers were asked questions related to resource constraints (ie, time and cost).

The virtual qualitative interviews were recorded and transcribed using features available through Microsoft Teams. Transcriptions were deidentified, and recordings were subsequently deleted. Google Sheets files from the Roses, Thorns, and Buds activity were also saved using a unique identification number for each participant. Following all interviews, data from transcriptions and Google Sheets were analyzed using descriptive qualitative analysis [33,34]. This approach was selected over a thematic qualitative analysis, as the questions focused primarily on program content and design and often requested discrete answers (eg, ways of incorporating home safety content and time spent on the program). Essentially, the questions elicited primarily surface-level responses, and the resulting data are better aligned to descriptive analysis rather than thematic analysis, in which underlying themes are identified [34]. The research team (ie, PI, co-investigators, and research assistants) developed a codebook based on the interview protocol, with codes reflecting each topic area. For instance, there was a code for home safety content in EBPP and subcodes for content provided and not provided. Similarly, there was a code for response to IVR use in EBPP and subcodes for positive, neutral, or negative sentiment. The interview transcripts were then coded by the PI using ATLAS.ti (ATLAS.ti Scientific Software Development GmbH). Through the descriptive analytic approach, similar ideas or responses were identified. Responses from the Roses, Thorns, and Buds activity were organized into common responses using Microsoft Excel. These findings were then shared with the research team and agreed upon before finalizing the results.

#### Parent Focus Groups

We sought to recruit 10 parents for focus groups to inform the program, with a goal of 5 parents per focus group. The target sample size was based on considerations for ideal focus group size, funding constraints, and study timeline. While we ideally would have included 12 parents across 2 focus groups [35], as with provider interviews, we sought to maximize the number of participants per focus group within our available resources, getting as close as possible to the sample size recommendations. Initially, the inclusion criteria for parents were as follows: at least one child aged 0-5 years, ability to read and speak English, access to the technology necessary to participate in a meeting using web-based conferencing software, and current or previous participation in an EBPP. We attempted to recruit parents through the EBPP providers who participated in qualitative interviews. However, after 2 months of attempts, we were unable to recruit any parents through EBPP providers and their agencies. We revised our inclusion criteria such that the criteria related to participation in an EBPP were eliminated. Using the new inclusion criteria, we recruited parents through an advertisement on the authors’ university’s Children and Family Programs website and weekly email communication.

Study procedures for parent focus groups closely mirrored those of provider interviews. Interested parents contacted the PI to confirm eligibility and sign up for a virtual focus group. Each focus group lasted approximately 60 minutes. Participants received a US $35 gift card as compensation. The focus group protocol was developed by the research team and was structured similarly to that of the EBPP providers’ interviews, with questions adapted for the parent experience (eg, asking about experiences learning about home safety instead of delivering home safety information). The goal of the initial questions regarding sources of information on home safety and interests was to better understand the context in which parents approached the topic of home safety; our primary interest was around the questions asking for feedback on the use of IVR and about ParentSHIELD-VR. Each focus group had 3‐4 participants. Analysis of qualitative data was conducted in the same manner as the provider interview data described above.

### Build: IVR Program Development

Storyboards were developed to provide the initial structure of the virtual environment and the room layout of a virtual apartment. Hazard identification and supervision tasks were also outlined and added to specific scenarios. For example, in a kitchen room, hazards may include scattered knives on a tabletop, a misplaced pot with boiling water on a stove with the handle sticking out, and rotten fruits. Based on detailed scenarios with hazard identification and supervision tasks and room layout descriptions, the development team created a fully immersive virtual environment (VE) using the game engine “Unity.” The VE consisted of a living room, kitchen, 2 bedrooms (parent and child), and a bathroom. Interactivity with virtual objects in the VE and supporting virtual hand-based 3D user interfaces (3DUI) were also added to the experience to simulate the real-world interactive experiences. Many of the interactions used Earnest Robot’s Auto Hand–VR interaction package, which enhanced the experience with automatic hand posing. The IVR experience was intended for use with the Meta Quest 3 mixed reality (MR) headset and controllers, a midtier VR system available on the market with a large user population. The full experience included a tutorial session, which taught participants how to travel in the virtual home and interact with virtual objects, followed by the training session. Users were tasked with identifying and resolving hazards in the VE. Within the training session, players received feedback via 3DUI upon attempting to address each hazard. See Figure 2 for screenshots of the VE. In the top panel of Figure 2 is an example of a firearm hazard in the primary bedroom. The middle panel depicts the kitchen in ParentSHIELD-VR. Participants can see the number of hazards that need to be resolved. Two hazards can be seen in Figure 2: a pot of boiling water and a knife (on the counter next to the stove) within reach. The bottom panel depicts an example of a supervision scenario in ParentSHIELD-VR. When the parent places the baby in the bathtub, they are presented with a situation in which the older child calls for them in another room. The parent is asked to select a response to this scenario.

**Figure 2.**
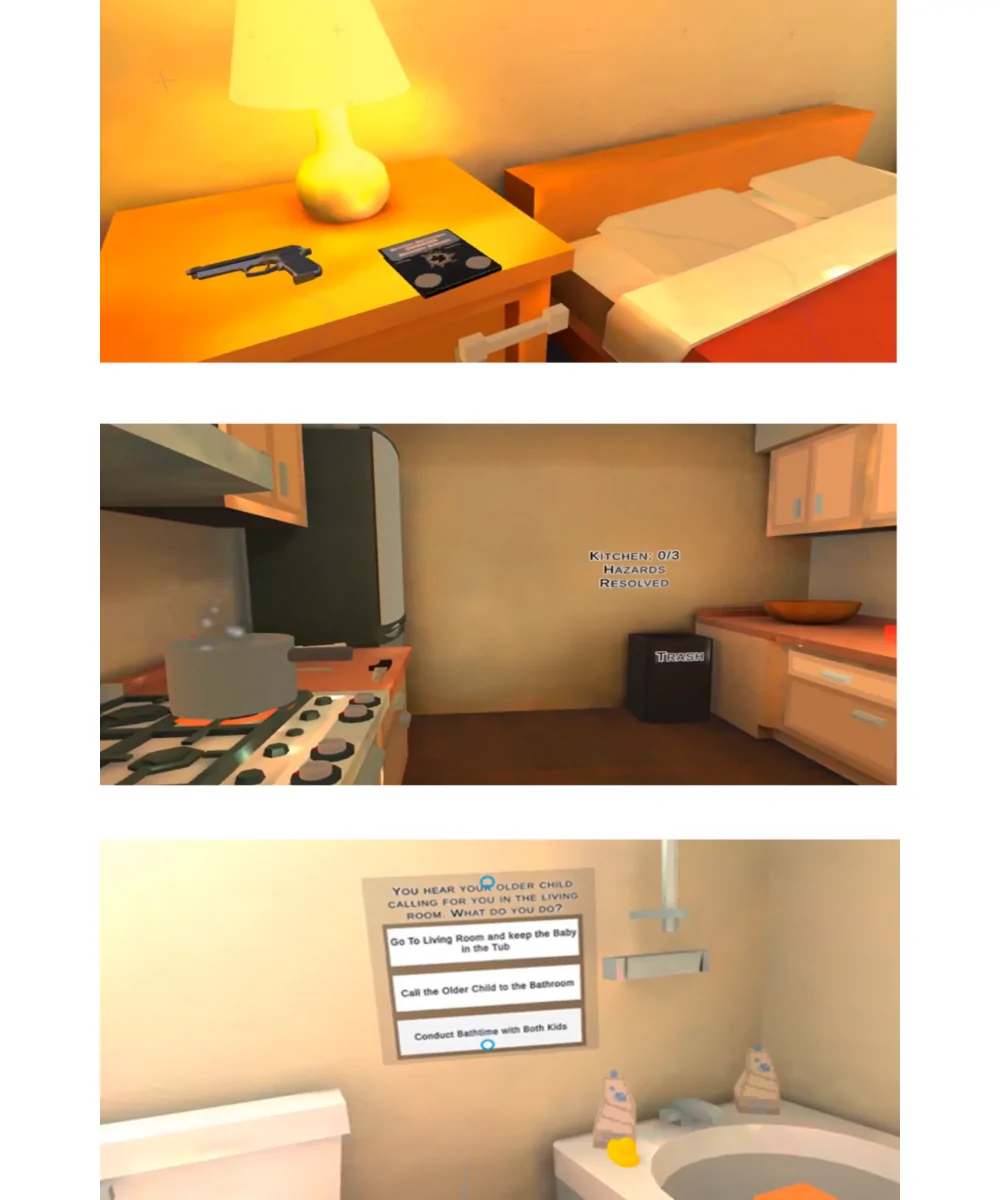
Screenshots from parent safe home immersive education and learning development–virtual reality (ParentSHIELD-VR).

### Test: Phase 2

#### Study Design and Procedures

Following prototype development, we sought to conduct prototype testing with 10 parents of children aged 0‐5 years, based on recommendations of 5‐10 individuals for usability testing [36,37]. Parents were recruited through flyers at local community organizations (eg, local libraries and social service agencies) and the authors’ university weekly email communications. Interested parents contacted the PI to confirm eligibility and schedule a research assessment at the university. At the research assessment, participants first completed a survey (pretest), then went through ParentSHIELD-VR, and finally completed a second survey (posttest). Measures in the pretest and posttest are described in more detail below. While we use the terms “pretest” and “posttest,” the study aims primarily focused on feasibility and usability data, which were collected at posttest. Secondarily, we aimed to collect data on the research process and to pilot measures related to home safety and child injury to be used in a future large-scale trial. When participants tested ParentSHIELD-VR, members of the research team were present to orient them to the headset, answer questions, and remain present for the session. Each research assessment took approximately 90 minutes, and participants were compensated with a US $99 gift card.

#### Measures

The pretest was administered using Qualtrics (Qualtrics International Inc) and included sociodemographic information, injury history, and a safety hazards knowledge assessment. Data from the latter 2 categories were collected as part of piloting measures in preparation for future studies examining injury outcomes. Sociodemographic items captured data on parents’ age, race, ethnicity, household income, and child’s age. Two items asked about child injury history in the past 3 months. First, parents were asked about the frequency of injuries that were treated at home: “In the past 3 months, how many accidental or unintentional injuries did your child have that you treated at home (eg, with an ice pack, bandages, etc)? This could be from a fall, scrape, burn, or any other injury that occurred and that you cared for at your home but did not require a visit to the doctor, hospital, or urgent care.” Then they were asked about the frequency of injuries that required treatment by a medical provider: “In the past 3 months, how many accidental or unintentional injuries did your child have that occurred at your home and required you to take them to seek care from a medical provider (eg, at the hospital, urgent care, or doctor’s office)?” Finally, participants’ knowledge of home safety hazards was assessed through a task in a physically simulated home environment in the authors’ university simulation center. A simulation space typically used for nursing home health simulations was transformed into a home with a young child and their family. Nine safety hazards were placed in the room by the research team. Participants were given 10 minutes to walk through the physical space and list the hazards in the Qualtrics survey.

The posttest included items on the research process, program usability, feedback on the program, and a safety hazards knowledge assessment. Specifically, items in the research process related to recruitment source, motivation to participate, and satisfaction with compensation. Program usability was assessed with the User Experience Questionnaire (UEQ) [38], a 26-item measure asking participants to rate the program using a 7-point Likert scale with bipolar anchors representing various elements of usability and experience. For instance, one item asks respondents to rate the program in terms of how annoying (1) or enjoyable (7) it is. Several items (3, 4, 5, 12, 23, 24, and 25) were reverse-coded such that 7 indicated the favorable side of the scale. However, note that some UEQ items do not necessarily have one favorable side (eg, fast to slow), and the open-ended items provided participants the opportunity to expand on the aspects of the program that they favored. Participants were given the Roses, Thorns, and Buds activity that was used in Phase 1, but it was completed via open-ended items in the Qualtrics survey. Finally, participants returned to the physical home simulation space and were given 10 minutes to assess the space for hazards.

#### Analytic Plan

Quantitative data (ie, sociodemographic information, the UEQ, and research process items) were summarized using descriptive statistics. Given the small sample size, UEQ items are presented as frequencies by category and range. As in Phase 1, qualitative data were analyzed using descriptive analysis. Data from the safety hazards knowledge assessment were examined in terms of the feasibility of using the physical simulation space to assess parents’ home safety knowledge rather than to assess knowledge itself. That is, we sought to determine whether assessing parents’ knowledge in this way was practical and to identify any barriers to collecting data in this way.

## Results

### Phase 1

#### EBPP Providers

Five EBPP providers (4 women and 1 man) were recruited for qualitative interviews. They represented 4 different EBPPs. Four of the 5 providers (80%) delivered programs that focused on parent-child interaction but incorporated home safety information; one provider delivered a program that had a dedicated home safety module along with parent-child interaction and other content. All providers incorporated home safety information in some way. The 4 providers who delivered an EBPP that did not have a dedicated home safety module all said that their approach to incorporating the content involved discussion of written information or videos, with some agencies providing parents with home safety devices. For example, one provider stated, “Yes, we do have some handouts, and then we give them to the parents....” Another provider said, “...we have a video we review. We provide a book that we review...as well as their worksheets and that kind of stuff in the book.” The one provider delivering the EBPP with a dedicated home safety module said she typically begins with that module if the family has been referred to the program based on allegations of safety concerns. When asked about home safety topics of interest to families, providers, interestingly, initially provided responses related to a range of safety topics, not necessarily related to in-home safety. For instance, 2 providers discussed families’ interest in learning how to avoid violence from strangers, one mentioned the importance of emotional safety, and one cited parents’ concerns about children’s physical health after a recent environmental crisis that occurred in their community in which toxins were released in the air. When prompted specifically about home safety topics that parents would benefit from, providers noted safe sleep, car seats, and “child proofing” (eg, cabinet locks and outlet covers). Four of the 5 providers in the sample reported limited use of technology for program delivery.

I have a computer to enter my [hours spent with families], but I don’t use it when I go to do my home visits. I just take papers.

[Provider C]

Some used their mobile phones or the parents’ phones to share videos during sessions (eg, “I take my phones and show them videos....”). While 2 providers reported that their EBPP had an app, they reported inconsistent use.

I mean, the app is only used with some people, honestly, if they want to see those handouts [digitally] or if they don’t want me to physically give them a copy, but we don’t use a lot of technology.

[Provider B]

All providers had an overall positive response to the idea of using IVR in EBPP sessions and thought it would be engaging to parents (eg, “I think that’ll be great,” “...it’s another great way to present the program and have parents explore,” “...it could be really powerful to do”). Questions and concerns about the use of IVR related to logistics (eg, who brings the headset and would Wi-Fi be required) and cost.

The families that we serve do not have a lot of access to things, so a lot of things may not have Wi-Fi. I have families that use only Text Now numbers, so they’re not even able to receive phone calls or see videos like that.

[Provider E]

This provider also noted that some parents may be “more receptive to it [IVR] than others just because of age.” Regarding ParentSHIELD-VR, providers also had an overall positive reception to the concept and name. One provider noted that the name may cause some parents to think of a popular movie franchise, but they did not think that was a negative thing. Providers suggested more realism in terms of the graphics (ie, higher-quality graphics) and the look of the VE. Of note, the rough prototype used to develop screenshots shared in Phase 1 featured a home setting with a bright, clean look, new appliances, and few imperfections. Providers suggested that there should be typical imperfections, such as piles of laundry, to make the house appear like a realistic family home and therefore more relatable to the average family they serve, with one provider especially emphasizing this issue.

My only worry would be like making it more real-ish for our parents because none of our parents has a clean—you know...they have more than just a knife on the counter.

[Provider B]

While the suggested time spent on the program ranged from 15 minutes to an hour, most providers said they would spend an hour. Two providers suggested incorporating the program into community events that their programs participate in, such as parent seminars that are held on a regular basis. Some suggested breaking up the experience across multiple sessions. Providers had questions about cost, and while the study participants did not make budgetary decisions in their respective organizations, 3 providers wondered if small grants or existing funds could be used to purchase IVR equipment.

...we’re pretty much federally funded. So, I guess it would have to go back to whether they deemed it something that we could use that’d be beneficial.

[Provider A]

Another said, “I think in the past we have had, like, a technology fund for a certain amount.”

#### Parents

In total, 7 parents (6 women and 1 man) were recruited for Phase 1 across 2 focus groups. Parents reported car seat installation, “baby proofing” their home, and safe sleeping as main safety topics of interest, with car seats mentioned most frequently. They reported a range of sources consulted when seeking home safety information, including professionals, their own social network, and internet sources. Professionals from whom parents received home safety information included pediatricians, health care professionals at the hospital postdelivery, and certified car seat installation technicians at local fire departments.

I think we received, like, a huge amount of pamphlets when he was born from the hospital, and I think we have gotten things from our pediatrician.

[Parent C]

They also sought information from friends and from other parents in social media parent groups. Parents reported searching for home safety topics using online search engines, YouTube (Google), apps, and ChatGPT (OpenAI). Online search engines (ie, Google) and YouTube were the most common sources used when seeking information about home safety on their own (ie, without a professional or another individual in their social network), with 3 participants reporting use of each of these approaches. One parent described the usefulness of online search engines, saying, “So usually when I’m needing information, it’s, like, quick and in the moment, and I’m just Googling.” Another parent said, “I Google a lot – like, a lot.” Some parents also reported that home safety topics appeared in their social media feeds, and it could be confusing to identify reputable sources and sort through conflicting information.

I feel like what comes to my mind initially is just Instagram. And sometimes it’s like a blessing and a curse. There’s a lot of information out there, so sometimes it makes me a little paranoid.

[Parent D]

I feel like I have to do a lot of research myself just to figure out how trust those online YouTube video and things on Instagram are. Very confusing and very time consuming too.

[Parent F]

Yeah, I think I would just echo I feel like I see a lot of conflicting information also on social media where there’s accounts that are like about safe sleep habits and like preventing SIDS, but then there’s accounts of ‘you can totally co-sleep safely; it’s your family’s choice,’ so it’s just very, like, differing opinions and not necessarily people who are coming from, like, a research mindset. It’s usually their own personal experience.

[Parent G]

Parents in the focus groups had an overall positive response to the use of IVR to learn about home safety and to ParentSHIELD-VR, describing it as “fantastic” and “something I wish I had when I was a first-time mom.” One parent pointed out the element of skill practice that IVR offers, saying, “I like that because I do learn better personally by doing.” As none of the parents had participated in an EBPP, they had suggestions for places where they would like to encounter the program, including prenatal visits, pediatrician visits, and use of the program on their own at home. They noted that first-time parents may be more receptive to the information and have more time to spend on it.

If you’re at the pediatrician’s office you have your kid there with you...But if you’re at the OB/GYN before you have a kid, that’s a different story.

[Parent E]

As with providers, parents thought that the program would be improved with more realism in terms of the graphics and the look of the VE. Regarding graphics, one parent said “...it looks like a Minecraft game.” That parent also commented on the look and feel of the VE saying, “It was also super clean...That is not realistic.” One parent suggested adding a “kid’s-eye view.” This parent said that she had been given advice to:

get on the kid’s level to see the hazards. So, is there a way in your program to get on the kid’s level and see not just at the adult standing level but...on the ground with the kids, so you’re at kid eye level...?

[Parent A]

One parent suggested adding a feature to assess their own home for hazards through augmented reality.

It would be amazing if you could maybe, like, do integrated reality as I’m looking around my actual kitchen and then you put, like, a virtual knife on the counter, similar to what you did, but it’s, like, my home, so then it means more to me.

[Parent B]

In terms of time spent on the program, parent responses ranged from 5 to 60 minutes, with most saying they would spend 20‐30 minutes on it. One parent noted that she would have been able to spend more time on such a program when her children were younger.

I would say 20 to 30 minutes would probably be my max, but that’s just ‘cause I have two kids so I don’t have a lot of extra time, but when they were little [sic], I could probably do, like, 30 to 45.

[Parent B]

One parent suggested participating in the program in segments rather than in one encounter.

### Phase 2

Following Phase 1, refinements were made to the program based on participant feedback, including the development of a tutorial, the addition of a child’s bedroom, and addressing comments about realism through the use of a more modest, relatable home environment (eg, dust and dirt in high traffic areas, miscellaneous objects on side tables, and modest appliances and fixtures). Five parents participated in Phase 2 prototype testing. These parents were recruited through flyers distributed via a social service organization (n=2, 40%), university email communications (n=2, 40%), and the local public school system (n=1, 20%). They identified as non-Hispanic White (n=2, 40%), non-Hispanic Black (n=1, 20%), Hispanic White (n=1, 20%), and non-Hispanic multiracial (n=1, 20%), with a mean age of 30 (SD 2.12) years. Most reported an annual household income of <US $50,000 (n=3, 60%). While we did not formally collect data on prior IVR use, the research team noted through informal conversations with participants that they reported little exposure to IVR, with some having never used it before and one having used it for job-related training. In terms of injury history, all participants reported at least one injury treated at home in the past 3 months, and one reported a single injury requiring treatment by a medical professional.

The results of the UEQ indicated that participants found the program to be highly creative (median 7, IQR 4.5-7), innovative (median 7, IQR 6.5-7), and organized (median 6, IQR 6-7). There was variability in ease of learning (median 5, IQR 3-6.5) and predictability (median 5, IQR 3-5.5). See Table 1 for UEQ results. Participants provided free text responses to the Roses, Thorns, and Buds activity. Elements of the program that participants liked included learning about home safety, the use of IVR to learn about home safety, and learning how to respond to hazards that may not be in their own home (eg, considering what to do with button batteries when you have not encountered them before). Elements that participants did not like included some program elements that did not function during testing (eg, doors that were difficult to open), experiencing motion sickness, time to become accustomed to the technology, and a lack of realism (eg, “some things don’t look too real which makes them hard to identify”). Regarding the lack of realism, in observation notes taken during assessments, research team members identified items in the VE that parents asked about, including cleaning products, bath soap bottles, and a button battery pack, that were difficult to identify. Finally, elements that participants wanted to add to the program were hazards outside the home (eg, pool) and an in-person component.

**Table 1.** UEQ^a^ results for Phase 2 participants (N=5). Frequencies are presented in the cells. For instance, on a scale of dull to creative (Item 3), 4 respondents selected a rating of 7.

UEQ item number and scale	Rating						
	1	2	3	4	5	6	7
1: Annoying to enjoyable	0	0	1	0	0	3	1
2: Not understandable to understandable	0	0	0	0	1	3	1
3: Dull to creative^b^	0	1	0	0	0	0	4
4: Difficult to learn to easy to learn^b^	0	1	0	1	1	1	1
5: Inferior to valuable^b^	0	1	0	0	1	2	1
6: Boring to exciting	0	1	0	1	1	2	0
7: Not interesting to interesting	0	0	0	0	0	4	1
8: Unpredictable to predictable	0	0	2	0	2	1	0
9: Fast to slow	0	1	2	1	1	0	0
11: Obstructive to supportive	0	0	0	0	1	3	1
12: Bad to good^b^	0	0	0	1	0	3	1
13: Complicated to easy	0	0	0	1	1	3	0
14: Unlikeable to pleasing	0	1	0	0	1	2	1
22: Impractical to practical	0	0	0	1	2	2	0
23: Cluttered to organized^b^	0	0	0	0	0	3	2
24: Unattractive to attractive^b^	0	0	0	1	0	3	1
25: Unfriendly to friendly^b^	0	0	0	0	0	3	2
26: Conservative to innovative	0	0	0	0	0	1	4

a UEQ: user experience questionnaire.

b Items were reverse coded.

As with Phase 1 parents, parents in Phase 2 were not recruited through EBPPs and had suggestions for alternative dissemination routes, including high school early childhood classes. Parents also provided general feedback on ParentSHIELD-VR and on research participation in an open-ended question. One participant said that she had learned about a new thing.

blinds and putting the strings up. My current blinds don’t have them but I can definitely see that causing issues for a small child.

[Parent I]

I’ve learned a lot about safety hazards through traditional methods...but this concept made me think about ways to show those hazards to new parents or caregivers....

[Parent K]

Finally, regarding research participation, a parent noted, “I learned that there is [sic]people looking after child safety, and ways to help parents achieve it, which is great.” Three parents specifically noted that the program was “fun,” “enjoyable,” and/or “cool” in their responses.

## Discussion

### Principal Findings

This proof-of-concept study found that an IVR home safety program was positively received by parents and EBPP providers, and they identified key areas for further development. While this is a small proof-of-concept study and more research is needed, this IVR approach shows promise as a child injury prevention strategy. Parents appreciated a unique opportunity for interactive and immersive learning on home safety, and EBPP providers saw the possibilities of increased engagement through IVR. Parents had a range of prior experience with and ease of learning in using IVR. Future work on ParentSHIELD-VR will incorporate approaches that bypass challenges in acclimating to the VE and reducing cognitive load. Prior research has found that, despite the many benefits of IVR, it can increase cognitive load, which has implications for learning outcomes [39,40]. This can be addressed with careful attention to design, including just enough details to maintain representational fidelity without overstimulating the user or requiring a great deal of cognitive processing [24]. For instance, in the virtual home environment in ParentSHIELD-VR, interior doors could remain open, eliminating the need for the user to open them. This could be done using additional interaction techniques based on the design of virtual hands. Future iterations of ParentSHIELD-VR will incorporate this approach.

In addition to cognitive load, other elements of parents’ interaction with IVR may impact their engagement with or outcomes associated with the program. First, prior experience using IVR could have an effect. Parents who have had the opportunity to acclimate to the VE and are more confident using the IVR equipment (ie, headset and controllers) may feel more comfortable engaging with the program. This could impact their experience of the program and their openness to learning, as has been found in prior research [41,42]. While we did not collect data on prior experience with IVR, this is an area for future research. Furthermore, cybersickness (ie, experiencing motion sickness, such as nausea and dizziness, during IVR use) may affect parents’ experiences with the program. One participant in our study reported motion sickness as a “thorn,” or an area of the program in need of revision. The estimated extent to which cybersickness affects IVR users varies, but some studies suggest that over half of users experience cybersickness when using head-mounted display-based IVR, and a greater proportion of women report it compared with men [43]. Cybersickness can be influenced by factors including postural instability, approach to moving in the VE (eg, use of joystick locomotion as opposed to real walking in the physical space), prior experience with IVR, and individual susceptibility to motion sickness [44-46]. For instance, use of teleportation and sitting (as opposed to standing) are approaches to moving in the virtual space that are associated with reduced risk of cybersickness [44]. Additionally, novice users and those who are susceptible to motion sickness in the physical realm are at increased risk for cybersickness symptoms [45,46]. Guidance on various options for moving through the VE (ie, real walking, joystick locomotion, and teleportation) was included in the ParentSHIELD-VR tutorial in an effort to reduce the risk of cybersickness. However, the implications for cybersickness may not have been made explicit. The primary cause of cybersickness in our prototype may be the constant virtual movement required to complete the tasks. We can mitigate this risk through 3 design changes. First, we can encourage users to choose teleportation instead of continuous locomotion. Teleportation is already available as an alternative movement option in the prototype, but the tutorial should explain its benefits more clearly and show users how to switch to it if they begin to feel sick. Second, we can apply a tunnel vision effect during continuous locomotion. Temporarily reducing the user’s field of view while they are moving can help minimize cybersickness. This technique has been available in the Unity VR template project and many commercial VR experiences involving virtual movement. Third, we can map the virtual environment to the available physical space. This would allow users to navigate the virtual environment by walking naturally in the real world, reducing their reliance on artificial locomotion.

ParentSHIELD-VR was designed to be implemented within the context of EBPP. Results of this proof-of-concept study shed light on potential issues related to implementation in this setting, which should be explored further. EBPP providers had questions about the costs of an IVR program. The providers in our study were not involved in budgetary and administrative decisions. However, they wondered who would be paying for the VR headset and noted that their organizations sometimes apply for small grants to purchase new technology. They also suggested implementing the program with parents who already own a VR headset. This could have the added benefit of engaging parents who are already familiar with and motivated to use IVR. Of note, some providers brought up concerns regarding lack of Wi-Fi in some families’ homes. ParentSHIELD-VR would come loaded on the VR headset, preventing the need for an internet connection. Ultimately, while we propose that this program could benefit all EBPP families, it may be the case that when EBPP agencies have limited technology available, ParentSHIELD-VR should be targeted to families who would most benefit. For instance, families who have been reported to child welfare for allegations of neglect related to safety practices have a clear need for home safety education. One of our provider participants noted that she begins with home safety education when working with such families. Also, families who are already engaging in IVR and therefore have already overcome the common barriers, such as cybersickness or difficulty learning how to operate the equipment, may be most receptive to the program and derive the most benefit from it. However, more work is needed to determine approaches for identifying families who would benefit most and the contexts in which IVR is most effective [47]. Research on virtual reality in mental health and behavioral health settings has found cost to be an implementation barrier, despite clinician or provider acceptance of the technology [48]. In one study, 2 substance use disorder treatment clinics shared a VR headset to save costs, but this resulted in inefficiencies in scheduling patients [48]. In mental health treatment, concerns around security and patient privacy have arisen as barriers to implementing telehealth or VR therapy [49]. While this has not been studied explicitly among EBPP providers, they may share these concerns, and future work should explore this potential barrier. There is also an indication that adoption of IVR in mental health treatment settings follows organizational norms around adopting innovative practices in general (ie, technological or otherwise), and some organizations may be slower to implement new practices than others [49,50]. The potential resource constraints associated with implementing IVR programs in EBPP are an area in need of research.

### Limitations

The findings in this proof-of-concept study should be interpreted in the context of several limitations. Phase 1 and Phase 2 participants were primarily from the Southeastern United States (94%), and there may be regional differences in preferences for safety and parenting information that would be identified with a sample from multiple geographic regions. Additionally, while Phase 2 parent participants represented a diverse set of races, ethnicities, and socioeconomic backgrounds, all identified as women. Gender differences may exist with regard to the receptivity of home safety education and interactions with technology. Relatedly, no sociodemographic information was formally collected on Phase 1 participants. Thus, while we have information on participant gender based on research team member interview notes, we cannot report detailed sociodemographic information that would assist in assessing sample representativeness for Phase 1. The study has limitations regarding sample size. In Phase 1, our sample sizes were slightly below recommended sizes for qualitative research due to funding constraints and challenges with recruitment, which may have resulted in some points not being identified, although studies have found that few new concepts are identified after the fifth or sixth interview [31,51]. The Phase 2 sample consisted of only 5 participants. While this is in line with conventional recommendations for usability testing using heuristic evaluation [36,37], we acknowledge the discussion regarding the need for larger sample sizes in usability testing [52,53]. Further testing (ie, beyond proof-of-concept) is needed to obtain expanded perspectives on the usability and acceptability of ParentSHIELD-VR. Finally, parents in the study were not recruited from EBPPs, as intended, which could limit generalizability to the population of EBPP participants. This was especially true with regard to Phase 1 parents, all of whom were ultimately recruited from the university environment. Data from evidence-based home visiting programs (a subset of EBPP) indicate that a substantial proportion of parents participating in these programs (21%) do not have a high school diploma, and 84% have children who are on public insurance [11]. Parents referred to EBPP are often (but not always) experiencing family stressors such as financial stress or violence in the home. These characteristics (ie, low socioeconomic status and family-level stressors) may bring unique challenges or perspectives that were not captured among our parent participants, who reported higher levels of income and education. While Phase 2 parents were not from EBPPs, 60% reported an annual family income of <US $50,000, and those parents may be similar to the average EBPP participant in that regard. Additionally, responses from Phase 1 providers often incorporated the ways they anticipated that parents might respond to the program or potential challenges, but we recognize that this is not a replacement for learning directly from parents in EBPP. Future research should include the voices of parents in EBPP, addressing recruitment challenges through strong collaborations with EBPP agencies.

### Conclusion

In this proof-of-concept study, ParentSHIELD-VR emerged as a potentially feasible way to engage parents of young children in home safety education. This study was proof-of-concept, and refinements and additional testing are needed to address user recommendations for the program and to assess its efficacy in addressing outcomes related to parent behavior and child injury. Our team has begun program refinements, including the use of improved graphics and clear labels to improve the realism of products in the VE. While ParentSHIELD-VR is in the early stages of development and testing, the approach has the potential to be translated to additional populations, such as parents of older children and teenagers. As children age, new hazards and concerns emerge, and these could be incorporated into a future iteration of the program. As one provider noted in Phase 1, many families have a “wide age range” between children, and they may be experiencing multiple developmental phases simultaneously. Future development and research could explore the potential benefits and opportunities of more recent technological advancements, such as the integration of AI into the IVR system through, for example, tailored, responsive feedback throughout the program to create a more adaptive and personalized training experience. IVR opens up a range of unique opportunities to engage parents in home safety education to prevent child injury.

## Abbreviations

3DUI: 3D user interface
CAMIL: cognitive affective model of immersive learning
COM-B: capability, opportunity, and motivation lead to behavior
EBPP: evidence-based parenting program
IVR: immersive virtual reality
MR: mixed reality
ParentSHIELD-VR: parent safe home immersive education and learning development–virtual reality
PI: principal investigator
UEQ: User Experience Questionnaire
VE: virtual environment
